# Relationship between disease activity level and physical activity in rheumatoid arthritis using a triaxial accelerometer and self-reported questionnaire

**DOI:** 10.1186/s13104-021-05666-w

**Published:** 2021-06-27

**Authors:** Yoichi Toyoshima, Nobuyuki Yajima, Tetsuya Nemoto, Osamu Namiki, Katsunori Inagaki

**Affiliations:** 1 Department of Orthopedics, Showa University School of Medicine, Shinagawa-ku, Tokyo, Japan; 2 Department of Rheumatology, Department of Medicine, Showa University School of Medicine, Shinagawa-ku, Tokyo, Japan; 3 Department of Healthcare Epidemiology, Graduate School of Medicine and Public Health, Kyoto University, Kyoto, Japan; 4 Center for Innovative Research for Communities and Clinical Excellence, Fukushima Medical University, Fukushima, Japan

**Keywords:** Accelerometer, Disease activity, Physical activity, Questionnaire, Rheumatoid arthritis

## Abstract

**Objective:**

This study evaluated the relationship between rheumatoid arthritis (RA) disease activity level and physical activity (PA) by using an accelerometer and self-reported questionnaire.

**Results:**

The cross-sectional study was part of a cohort study designed to determine disease activity is associated with PA in RA patients. We classified patients with a Disease Activity Score 28-erythrocyte sedimentation rate (DAS28-ESR) of less than and higher than 3.2 into the low-disease-activity (LDA) group and moderate/high-disease-activity (MHDA) group, respectively. We measured the wear time, time of vigorous-intensity PA, moderate-intensity PA, light-intensity PA, and sedentary behavior per day using a triaxial accelerometer. 34 patients were included in the study. The accelerometer-measured moderate-to-vigorous PA (MVPA) was 17.2 min/day and 10.6 min/day in the LDA group and MHDA group (p < 0.05), respectively. There was no significant association between RA disease activity level and accelerometer-measured PA with adjustment for age and Functional Assessment of Chronic Illness Therapy-Fatigue score. There was no correlation between accelerometer-measured MVPA and self-reported MVPA in the MHDA group, but these factors were correlated in the LDA group (rs = 0.57, p < 0.05). In conclusion, no significant association was noted between RA disease activity level and accelerometer-measured PA.

**Supplementary Information:**

The online version contains supplementary material available at 10.1186/s13104-021-05666-w.

## Introduction

Rheumatoid arthritis (RA) is a systemic, progressive, chronic disease. Body functions in patients with RA are reduced compared with healthy individuals because of joint problems, systemic lesions, and fatigue [1].

Physical activity (PA) has a positive effect on preventing several chronic diseases and can reduce all-cause mortality [2, 3] Evaluating PA and rehabilitation in RA patients can help prevent heart diseases, diabetes, hypertension, depression, and osteoporosis and are associated with an improved low mortality rate [4–6]. Comparing patients with RA and healthy subjects was reported that the amount of PA was significantly lower in patients with RA than in controls [7]. In several studies evaluating moderate-to-vigorous PA (MVPA) using accelerometers in patients with RA, RA patients showed lower MVPA than healthy subjects [7–9]. Hernandez-Hernandez et al. evaluated the relationship between delta MVPA and delta PA by using an accelerometer and found that PA increased with the improved disease activity [9, 10]. However, other studies using accelerometer reported that disease activity levels did not significantly correlate with PA [8, 11]. Patients with RA are more likely to feel fatigued and depression due to high disease activity, resulting in affected PA [12, 13]. There have been no reports evaluating PA and Disease activity adjusted for fatigue in the patient RA.

Previously, PA was converted into metabolic equivalents (METs) by using a self-reported questionnaire and was evaluated [14]. Recently, an accelerometer has attracted some attention as a valuable tool for evaluating PA [15–18]. The triaxial accelerometer can evaluate changes in activity levels and separate sedentary time and active time [19–21]. Population-based research, such as the National Health and Nutrition Examination Survey, have used accelerometers for PA measurements rather than conventional methods [22]. By contrast, some studies examining the relationship between RA disease activity and PA levels indicated a correlation between decreased disease activity and increased PA based on a self-reported questionnaire [23, 24].

As a method for evaluating PA levels in patients with a substantial amount of sedentary behavior (SB), no report has directly compared and examined the accuracy of the triaxial accelerometer and a self-reported questionnaire based on the disease activity level of patients with RA.

This study aimed to evaluate the relationship between RA disease activity level and the amount of PA measured using a triaxial accelerometer and self-reported questionnaire. We hypothesized a significant correlation between disease activity levels and PA and between self-reported PA and accelerometer-measured PA.

## Main text

### Materials and methods

The cross-sectional study was part of a cohort study designed to determine disease activity is associated with PA in RA patients. From November 22, 2015 through December 31, 2016, consecutive patients who visited their rheumatologist at the outpatient clinic of the Showa University Hospital in Tokyo, Japan were invited to participate. The eligibility criteria were as follows: (1) fulfillment of the American Rheumatism Association 1987 revised Criteria for RA [25] and (2) age of 20–65 years at consent acquisition. The excluded patients were judged ineligible by the research doctor for the following reasons: (1) bedridden/requiring a wheelchair, (2) dementia, and (3) lower limb deficiency that requires prosthesis for walking. A study with 50 participants was designed on our preliminary research, and 41 patients agreed to wear an accelerometer. However, seven patients were excluded (Additional file 1). Finally, 34 patients were included in the study. Disease activity was measured using the Disease Activity Score 28-erythrocyte sedimentation rate (DAS28-ESR) [26]. We classified patients with a DAS28-ESR of less and more than 3.2 into the low-disease-activity (LDA) and moderate/high-disease-activity (MHDA) groups, respectively [27]. Of the 34 patients, 20 were classified in the LDA group and 14 in the MHDA group.

The primary endpoint was PA evaluated using a triaxial accelerometer. The secondary endpoint was PA evaluated using the International Physical Activity Questionnaire (iPAQ) [28, 29].

PA was assessed using the triaxial accelerometer Active Style Pro HJA-750C (Omron Healthcare, Kyoto, Japan). We measured the wear time, time of vigorous-intensity PA (VPA), moderate-intensity PA (MPA), light-intensity PA (LPA), SB, and the number of steps per day using this device. MET-based cutoffs were used to define the intensity of each activity as follows: ≤ 1.5 METs for SB, 1.6–2.9 METs for LPA, and ≥ 3 METs for MVPA [30, 31]. The participants wore the accelerometer on their waist for seven consecutive days while they were awake. They did not wear the accelerometer when engaging in water-involving activities, such as swimming and showering. Records obtained when the accelerometer was worn for at least 10 h/day were considered valid, and data were considered as “nonwear” when acceleration signals were not observed continuously for more than 60 min [32]. The CSV data files of the accelerometer were downloaded using Omron health management software BI-LINK for PA Professional Edition ver. 1.0. The files were processed using custom software (i.e., a custom-written macro program for compiling data).

A short iPAQ form was used to determine PA during leisure time, domestic work, paid or unpaid work, and transportation [28, 29, 33, 34]. The patients were questioned regarding the following three specific types of PA, in which they participated at any time during their daily routine: walking, moderate-intensity activity, and vigorous-intensity activity. Scores for each type of activity were calculated by summing the scores for duration and frequency. Published guidelines for data processing and analysis of IPAQ data were used (available from: http://www.ipaq.ki.se). Comorbidities were evaluated using the Charlson comorbidity index [35]. At the outpatient clinic visit, the subjective physical function was assessed using the Modified Health Assessment Questionnaire (mHAQ) [36]. The health-related quality of life was measured using the Medical Outcomes Study (MOS) 12-item Short-Form Health Survey (SF-12) [37]. The Functional Assessment of Chronic Illness Therapy-Fatigue (FACIT-F version 4) questionnaire was used to calculate the level of fatigue [38, 39]. The Center for Epidemiologic Studies Depression Scale (CES-D) was used to assess depressive symptoms [40]. Medical data (e.g., body mass index [BMI], disease duration, and medication history) of patients with RA were collected from medical charts. Radiological evaluation of the lower extremity involved six classes of the Larsen classification [41].

Statistical analysis was performed using JMP® 13 software (SAS Institute Inc., Cary, NC, USA). Continuous data were expressed as means with standard deviations (SD) or medians with interquartile ranges (IQR). The normal distributions of each dataset were evaluated by performing the Shapiro–Wilk test. Spearman’s rank-sum test was used to assess the correlation. To assess the association between RA disease activity level and physical activity, multilinear regression analysis with adjustment for age and the FACIT-F score was performed (regression coefficient – 2.97; 95% CI: − 8.46 to 2.52; p = 0.28). For all analyses, statistical tests were two-sided, and significance was defined as p < 0.05.

## Results

The baseline characteristics of the study patients was presented (Additional file 2). Significant differences were noted in the mHAQ scores, swollen joint count-28, tenderness joint count-28, C-reactive protein level, ESR, and MMP-3 level of the groups. The FACIT-F score was significantly higher in the MHDA group than the LDA group (p = 0.003).

The mean (SD) values of the variables of subjective measures (i.e., SF-12) for the LDA and MHDA groups was shown (Additional file 3). For all items, except emotional role in the SF-12, the scores were significantly higher for the LDA group than the MHDA group.

For all RA patients, the median accelerometer wear time, median SB time, and median MVPA were 696.0 min/day (IQR, 630.9–743.2), 424.6 min/day (IQR, 386.9–458.6), and 14.9 min/day (IQR, 10.5–20.8), respectively.

Table 1 presents time spent in objectively measured PA in the LDA and MHDA groups. The median accelerometer MVPA was 17.2 min/day (IQR, 13.5–21.8) and 10.6 min/day (IQR, 9.5–15.7) in the LDA group and MHDA group, respectively. Most of the wear time was spent during SB. The analysis showed that MVPA was significantly lower in the MHDA group than in the LDA group (p = 0.018). A significant difference in SB between the two groups was not noted (p = 0.015).

**Table 1:** Comparison of PA in the LDA and MHDA groups

Variables	LDA group (n = 20)	MHDA group (n = 14)	p-value
	Median (IQR)	Median (IQR)	
Accelerometer (min/day)			
Wear time	764.6 (643.4–724.8)	739.1 (605.6–744.9)	0.13
MVPA	17.2 (13.5–21.8)	10.6 (9.5–15.7)	0.28
LPA	256.2 (235.2–261.9)	256.2 (225.6–276.8)	0.15
SB	493.6 (389.7–434.0)	468.0 (373.8–458.6)	0.15
Steps/day	6425 (5892–7645)	4877 (4190–7039)	0.37
iPAQ (METs/min/week)			
Total PA score	2222.3 (2882.9)	1004.9 (878.8)	0.47
VPA	348 (1287.7)	171.4 (641.4)	0.91
Moderate PA score	702.6 (1281.2)	212.6 (322.5)	0.55
MVPA score	1050.6(1284.4)	384.0(963.9)	0.44
Walking PA score	1171.7 (933.9)	620.9 (310.7)	0.13

*LDA* low disease activity, *MHDA* moderate/high disease activity, *SB* sedentary behavior, *LPA* light-intensity physical activity, *MVPA* moderate-to-vigorous physical activity, *VPA* vigorous physical activity, *MPA* moderate physical activity, *IQR* interquartile range, *METs* metabolic equivalents, *iPAQ* International Physical Activity Questionnaire, *PA* physical activity

**Fig. 1:**
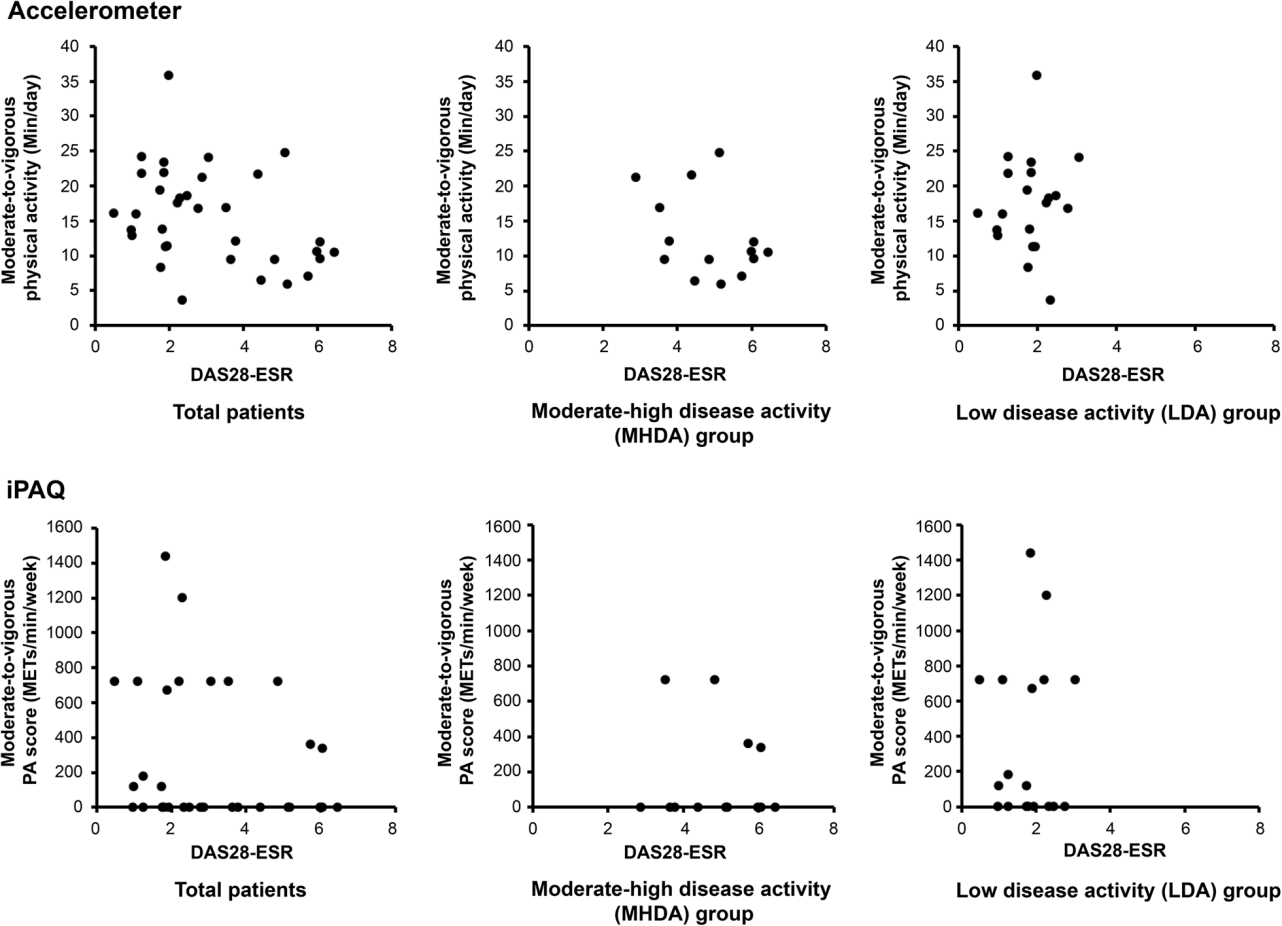
The relationship between DAS28-ESR and moderate-to-vigorous physical activity (MVPA) obtained from an accelerometer and iPAQ. There was no association between DAS28-ESR and accelerometer-measured MVPA in total patients (rs = 0.13, p = 0.03), LDA group (rs = 0.15, p = 0.31), or MHDA group (rs = 0.01, p = 0.59). Furthermore, there was no association between DAS28-ESR and self-reported MVPA in total patients (rs = 0.05, p = 0.19), LDA group (rs = 0.01, p = 0.92), and MHDA group (rs = 0.01, p = 0.8). DAS28-ESR, Disease Activity Score 28-joint count erythrocyte sedimentation rate

RA disease activity was not associated with the MVPA score in the multilinear regression analysis adjusted for age and FACIT-F score (regression coefficient – 2.97; 95% CI: − 8.46 to 2.52; p = 0.28). No association was observed between DAS28-ESR and accelerometer-measured PA in any patients with RA, LDA group, or MHDA group. Furthermore, there was no association between DAS28-ESR and self-reported PA for any group (Fig. 1). We assessed the correlation between the accelerometer-measured and self-reported PA evaluated in all RA patients, the LDA group, and the MHDA group (Table 2). The MVPA measured using the accelerometer and the MVPA score identified using the iPAQ showed a relative correlation for the LDA group (rs = 0.57, p < 0.05) but not for all the patients or the MHDA group.

**Table 2:** Spearman’s correlations between iPAQ score and accelerometer measurement

	Total patients (n = 34)			LDA group (n = 20)			MHDA group (n = 14)		
	Accelerometer								
	MVPA	LPA	Steps/day	MVPA	LPA	steps/day	MVPA	LPA	Steps/day
iPAQ									
MVPA	0.26	0	0.01	0.57*	0.05	0.07	– 0.39	− 0.12	− 0.28
Walking PA score	0.25	0.17	0.58**	0.06	0.01	0.51*	0.46	0.37	0.64*

*LDA* low disease activity, *MHDA* moderate/high disease activity, *iPAQ* International Physical Activity Questionnaire, *PA* physical activity, *LPA* light-intensity physical activity, *MVPA* moderate-to-vigorous physical activity; *p < 0.05, **p < 0.001

## Discussion

In this study, we measured PA using a triaxial accelerometer and a self-reported questionnaire in patients with RA. The median time of the accelerometer-measured MVPA was 17.2 min/day and 10.6 min/day for the MHDA and LDA groups, respectively. No significant association was noted between the RA disease activity level and accelerometer-measured PA after adjusting for age and fatigue. No correlation was noted between the accelerometer-measured and self-reported PA for total RA patients or the MHDA group, but these two factors were correlated in the LDA group (rs = 0.5).

The results revealed no significant association between RA disease activity level and accelerometer-measured PA. Thus, disease activity in patients with RA and PA may not be related. The results of this study differed from previous studies that reported a relationship between RA disease activity and self-reported PA [9, 24]. The findings can be attributed to by several possible explanations. First, a different method was used to assess outcomes. PA was evaluated using a self-report questionnaire in previous studies [15, 16, 22], whereas we measured PA using a triaxial accelerometer. The result of the relationship between disease activity and PA might have been different because self-reported PA and the more accurate accelerometer-measured PA results are different. Second, the DAS28-ESR may not be suitable for evaluating disease activity related to PA in patients with RA. Most of the joints evaluated using the DAS 28-ESR are upper extremity joints; only two extremity joints are assessed. Therefore, the DAS28-ESR, which evaluates 28 joints primarily located in the extremities, may not be suitable as an evaluation method to assess RA disease activity affecting PA, such as moderate and vigorous movement [8, 42, 43]. To evaluate the relationship between disease activity and PA for patients with RA, the joint destruction of both the lower and upper extremity joints should be evaluated [44]. Third, we evaluated the relationship between disease activity and PA, adjusting for fatigue. Fatigue is a common symptom among RA patients [13]. Fatigue is associated with disease activity in RA, and also with PA in RA patients [13, 38]. Disease activity, fatigue, and PA might be associated in RA patients. For comprehensive health management of RA patients, it may be important not only to improve disease activity but also the treatment of fatigue by cognitive-behavioral therapy and improvement of physical function by physical exercise [45, 46].

The MVPA evaluated by self-report and accelerometer may not be correlated in moderate/high-disease-activity RA patients. Our results differ from previous reports where self-reported PA was relatively consistent with accelerometer-measured PA among healthy subjects [29, 47]. There are several possible reasons for this finding. First, the iPAQ only measures MVPA sustained for more than 10 min. However, the triaxial accelerometer can measure short-term MVPA lasting for fewer than 10 min. Therefore, the PA results obtained from the two methods were different [48, 49]. Second, MVPA is overestimated, and SB is underestimated in the self-reported questionnaire compared with that in the accelerometer [50–52]. Moderate PA might be overestimated by self-reported PA compared with the accelerometer-measured PA in the moderate/high-disease-activity RA patients.

To the best of our knowledge, this is the first study to use a triaxial accelerometer and self-reported questionnaire for a direct comparison between habitual PA for LDA and MHDA patients with RA. Further, disease activity and PA were measured in continuously sampled patients in actual clinical practice. Finally, fatigue was adjusted as a relevant confounding factor that could affect the association between RA disease activity and PA [53–55]. In our study, patients with RA had limited time of MVPA despite very low levels of disease activity and disability, thus suggesting the possibility of additional factors influencing MVPA levels in this population.

In conclusion, no significant association was noted between RA disease activity level and accelerometer-measured PA after adjusting for age and fatigue. The data will be useful for epidemiological studies and the self-health management of RA patients.

## Limitations

This study has several limitations. First, because this is a cross-sectional study, we were unable to conclusively determine causality. However, we believe that disease activity in patients with RA reduces PA levels, which is biologically plausible, but reverse causality remains possible. Second, the sample size was small and was not an even set of subsamples. We could not control disease specificities (i.e., organ damage or disease activity affecting PA). Correlations may become stronger with smaller observation groups. This was a pilot study, and we will perform a further study with more subjects in the future.

## Supplementary Information

**Additional file 1:** A flowchart of our participants’ selection.

**Additional file 2:** Title of data: Characteristics of the participants.

**Additional file 3:** Title of data: Subscale of SF-12 of LDA and MHDA groups.

## Data Availability

The datasets used and/or analyzed during the current study are available from the corresponding author on reasonable request.

## Abbreviations

RA: Rheumatoid arthritis
PA: Physical activity
DAS28-ESR: Disease Activity Score 28-erythrocyte sedimentation rate
LDA: Low disease activity
MHDA: Moderate/high disease activity
MVPA: Moderate-to-vigorous PA
SB: Sedentary behavior
iPAQ: International Physical Activity Questionnaire
VPA: Vigorous-intensity PA
MPA: Moderate-intensity PA
LPA: Light-intensity PA
mHAQ: Modified Health Assessment Questionnaire
MOS: Medical Outcomes Study
SF-12: 12-Item Short-Form Health Survey
FACIT-F: Functional assessment of chronic illness therapy-fatigue
CES-D: Center for Epidemiologic Studies Depression Scale
BMI: Body mass index
ACPA: Anticitrullinated protein antibodies
RF: Rheumatoid factor
SD: Standard deviations
IQR: Interquartile ranges
