# Supplementary material for: Relationship between disease activity level and physical activity in rheumatoid arthritis using a triaxial accelerometer and self-reported questionnaire
Source: BMC Res Notes. 2021 Jun 27;14:242. doi: 10.1186/s13104-021-05666-w (PMC8237436; doi:10.1186/s13104-021-05666-w)
Supplement: Supplementary file 1 — Additional file 1: A flowchart of our participants’ selection. [file 13104_2021_5666_MOESM1_ESM.docx]

**Additional file 1**: A flowchart of our participants’ selection
