# Supplementary material for: Relationship between disease activity level and physical activity in rheumatoid arthritis using a triaxial accelerometer and self-reported questionnaire
Source: BMC Res Notes. 2021 Jun 27;14:242. doi: 10.1186/s13104-021-05666-w (PMC8237436; doi:10.1186/s13104-021-05666-w)
Supplement: Supplementary file 2 — Additional file 2: Title of data: Characteristics of the participants. [file 13104_2021_5666_MOESM2_ESM.docx]

**Additional file 2**: Characteristics of the participants

|  | LDA group (n = 20) | MHDA group (n = 14) | Missing | p-value |
| --- | --- | --- | --- | --- |
|  |  |  |  |  |
| DAS28-ESR | <3.2 | ≥3.2 |  |  |
|  |  |  |  |  |
| Age (years) |  |  |  |  |
| mean (SD) | 54.6 (6.7) | 51.6 (8.0) | 0 | NS |
| Sex |  |  |  |  |
| Female: n (%) | 19 (95) | 10 (71) | 0 | NS |
| Body mass index |  |  |  |  |
| mean (SD) | 21.4 (4.0) | 19.5 (5.0) | 0 | NS |
| Charlson comorbidity index | 0.05 (0.2) | 0.07 (0.2) | 0 | NS |
|  |  |  |  |  |
| Corticosteroids |  |  |  |  |
| n (%) | 4 (20) | 4 (29) | 0 | NS |
| Biological DMARDs |  |  |  |  |
| n (%) | 9 (45) | 7 (35) | 0 | NS |
| Disease duration |  |  | 0 |  |
| <1y, n (%) | 0 (0) | 2 (14) |  | NS |
| 1y–5y, n (%) | 6 (30) | 3 (21) |  |  |
| ≧5y, n (%) | 14 (70) | 9 (64) |  |  |
| Surgery related orthopedics |  |  |  |  |
| One or over, n(%) | 3 (15) | 2 (14) | 0 | NS |
|  |  |  |  |  |
| ACPA positivity (≥7 U/mL) |  |  |  |  |
| n (%) | 14 (70) | 13 (93) | 0 | NS |
| RF positivity (≥15 IU/mL) |  |  |  |  |
| n (%) | 14 (70) | 13 (93) | 0 | NS |
|  |  |  |  |  |
| mHAQ | 0.12 (0.25) | 1.6 (1.4) | 0 | p < .001 |
| Swollen joint count: 28 | 0.55 (1.2) | 5.99 (5.45) | 0 | p < .001 |
| Tender joint count :28 | 0.35 (0.59) | 4.6 (4.6) | 0 | p < .001 |
| Radiographic evaluation |  |  | 0 |  |
| <Larsen II, n (%) | 11 (55) | 12 (86) |  | NS |
| ≥Larsen III, n (%) | 7 (35) | 2 (14) |  |  |
| C-reactive protein [mg/dL] |  |  |  |  |
| mean (SD) | 0.13 (0.15) | 2.64 (3.77) | 0 | p < .001 |
| ESR (mm/hour) |  |  |  |  |
| mean (SD) | 10.6 (7.2) | 29.9 (18.2) | 0 | p < .001 |
| MMP-3 (ng/mL) |  |  |  |  |
| mean (SD) | 55.3 (50.8) | 334.8 (322.0) | 0 | p < .001 |
|  |  |  |  |  |
| FACIT-F score |  |  |  |  |
| mean (SD) | 124.4 (16) | 104.3 (20) | 0 | p < .01 |

LDA, Low disease activity; MHDA, moderate/high disease activity; NS, non-significance; statistical analysis by Welch two-sample t test, Wilcoxon rank-sum test, Chi square test or Fisher’s exact test. DAS28-ESR, Disease Activity Score 28-joint count erythrocyte sedimentation rate; DMARDs, disease-modifying antirheumatic drugs; ACPA, anticitrullinated protein antibodies; RF, rheumatoid factor; FACIT-F, Functional Assessment of Chronic Illness Therapy-Fatigue; mHAQ, modified health access and quality index.
