# Supplementary material for: Relationship between disease activity level and physical activity in rheumatoid arthritis using a triaxial accelerometer and self-reported questionnaire
Source: BMC Res Notes. 2021 Jun 27;14:242. doi: 10.1186/s13104-021-05666-w (PMC8237436; doi:10.1186/s13104-021-05666-w)
Supplement: Supplementary file 3 — Additional file 3: Title of data: Subscale of SF-12 of LDA and MHDA groups. [file 13104_2021_5666_MOESM3_ESM.docx]

**Additional file 3.** Subscale of SF-12 of LDA and MHDA groups

|  | LDA (n = 20) | MHDA (n = 14) |  |
| --- | --- | --- | --- |
| variables | Mean (SD) | Mean (SD) | p-value |
| SF-12 |  |  |  |
| Physical functioning | 49.1 (11.8) | 33.0 (17.6) | .005 |
| Role: Physical | 44.6 (9.2) | 30.7 (14.5) | .004 |
| Body pain | 45.6 (7.6) | 31.1 (12.8) | .002 |
| General Health Perception | 53.5 (9.4) | 38.3 (10.6) | p < .001 |
| Vitality | 50.3 (7.3) | 42.4 (10.5) | .02 |
| Social Functioning | 49.7 (10.1) | 41.1 (13.2) | .01 |
| Role: Emotional | 47.2 (9.3) | 40.2 (14.6) | NS |
| Mental Health | 49.7 (7.4) | 41.9 (10.7) | p < .001 |
| Data expressed as mean and standard deviation (SD). LDA, Low disease activity; MHDA, moderate/high disease activity; SF-12, 12-item Short-Form Health Survey; *p < 0.01 by the Wilcoxon rank-sum test. | | | |
